# A Chatbot-Based Version of a World Health Organization–Validated Intervention (Self-Help Plus) for Stress Management in Pregnant Women: Protocol for a Usability Study

**DOI:** 10.2196/53891

**Published:** 2025-08-01

**Authors:** Silvia Rizzi, Valentina Fietta, Lorenzo Gios, Stefania Poggianella, Maria Chiara Pavesi, Chiara De Luca, Debora Marroni, Claudia Paoli, Anna Gianatti, Barbara Burlon, Vanda Chiodega, Barbara Endrizzi, Angela Giordano, Francesca Biagioli, Veronica Albertini, Marianna Purgato, Corrado Barbui, Chiara Guella, Erik Gadotti, Stefano Forti, Fabrizio Taddei

**Affiliations:** 1 Digital Health Research, Centre for Digital Health & Wellbeing, Fondazione Bruno Kessler Trento Italy; 2 Department of General Psychology, University of Padova Padova Italy; 3 Istituto Pavoniano Artigianelli Trento Italy; 4 Operating Unit of Psychology, Healthcare Trust of the Autonomous Province of Trento Trento Italy; 5 Transmural Obstetric Gynecological Department, Healthcare Trust of the Autonomous Province of Trento Trento Italy; 6 World Health Organization Collaborating Centre for Research and Training in Mental Health and Service Evaluation, Department of Neurosciences, Biomedicine and Movement Sciences, Section of Psychiatry, University of Verona Verona Italy

**Keywords:** stress management, promoting well-being, pregnancy, eHealth, mobile health, mHealth, mobile apps, development, usability, user-centered design, chatbot, multifaceted, woman, transformations, well-being, psychological health, healthy lifestyle, digital solutions, psychological

## Abstract

**Background:**

Pregnancy is a complex period involving significant physical, mental, and social changes in a woman’s life, affecting her psychological well-being. According to the literature, anxiety, stress, and depression are common symptoms among pregnant women. Promoting a healthy lifestyle with a focus on mental health is essential. In this context, digital solutions such as coaches on smartphones are emerging as valuable tools to support the psychological well-being of pregnant women without existing disorders.

**Objective:**

This study aims to present the research protocol of a pilot study designed as a proof-of-concept investigation. The study evaluates the feasibility, acceptability, and utility of an acceptance and commitment therapy–based stress management mobile app. The primary objective is to explore the feasibility of using a coach, ALBA (A Well-Being Assistant), developed within the TreC Ricerca app, to promote women’s psychological well-being during pregnancy through 5 sessions based on acceptance and commitment therapy. The pre- and postintervention effects on psychological well-being will also be explored as a secondary objective, serving as a proxy for the potential impact of the intervention.

**Methods:**

The study serves as a proof-of-concept investigation, where a small sample size (N=50) is deemed adequate to fulfill the study’s objectives. Participant recruitment will be conducted among pregnant women affiliated with the pregnancy care services of the Azienda Provinciale per i Servizi Sanitari di Trento, using a convenience sampling approach. ALBA will interact with the participating women for 6 weeks, between the 14th and 26th weeks of gestation. Specifically, there will be 1 session per week, which the woman can choose, to allow more flexibility regarding her needs, supplemented by ALBA-supported exercises to be performed between sessions. This study adopts a mixed methods approach, combining quantitative and qualitative data collection and analysis. Usability and engagement are assessed using the System Usability Scale, Chatbot Usability Questionnaire, User Engagement Scale-Short Form, and the User Mobile Rating Scale. Moreover, other quantitative outcome measures include levels of stress, anxiety, depression, emotional regulation, psychological flexibility, coping strategies, self-efficacy, and overall well-being, along with qualitative data from semistructured interviews. Finally, the analysis of the data gathered in this study will primarily adopt descriptive statistics and a text mining approach, focused on evaluating the attainment of the study objectives and changes over experimental time.

**Results:**

The psychoeducational approach aims to yield notable outcomes regarding the usability and engagement of women with ALBA. Furthermore, an anticipated enhancement in psychological well-being and quality of life is expected.

**Conclusions:**

Existing literature indicates a preference among women in the perinatal period for online support, highlighting the potential of digital interventions to address barriers related to social stigma and seeking assistance. In this context, ALBA emerges as a valuable resource, providing consistent psychoeducational support for women throughout pregnancy.

**International Registered Report Identifier (IRRID):**

PRR1-10.2196/53891

## Introduction

### Overview

Despite the increasing awareness of mental health issues and concerted efforts, a significant proportion of individuals with poor mental well-being do not receive prevention actions or appropriate care [1,2]. This treatment gap is influenced by various factors, including financial constraints, limited availability of services, and societal stigma, particularly for populations susceptible to stressful situations [3,4], including pregnancy.

The pregnancy period is characterized by essential transformations in the woman, with relevant impact on her physical, mental, and social well-being. How a woman adapts to these changes can affect her quality of life (QoL) and psychological well-being. The literature highlights that various psychological symptoms can be commonly experienced during pregnancy, ranging from anxiety, stress, and depression [5-9].

To date, psychoeducational interventions that promote women’s psychological well-being during pregnancy are scarce and tend to focus primarily on subgroups of women with psychiatric symptomatology, such as perinatal depressive disorder [5,10]. Evidence of the effectiveness of psychological interventions targeting pregnant women is increasing [11], but access to support services still presents several challenges. A considerable proportion of women face geographic barriers, with specialized centers often far from their homes [12,13]. In addition, a shortage of qualified staff, such as psychologists, further limits access to specialized care [12,13]. The stigma associated with mental health problems can also prevent women from seeking psychological support in these circumstances [12,13].

The overall strategy adopted by the World Health Organization (WHO) for mental health promotion is based on principles of inclusiveness and scalability for the public and susceptible populations [14]. WHO promotes inclusive mental health by aligning with human rights, ensuring cultural sensitivity, engaging diverse stakeholders, providing accessible and tailored services, and addressing social determinants to reduce disparities [15]. Within this framework, special attention is paid to the potential role of digital technologies in promoting psychological well-being, as it can be an optimal solution [16]; women can access it from anywhere and at any time, thus remaining flexible to their needs and reducing barriers related to service availability, social stigma, and seeking help [17].

The study described in this protocol is in line with WHO strategies and aims to assess the acceptability and feasibility of an intervention delivered through digital tools to promote psychological well-being. To the best of our knowledge, the intervention, originally validated by WHO and named Self-Help Plus (SH+), has never been used on this study’s target population, pregnant women.

SH+ is a low-intensity intervention developed by a multidisciplinary and international working group [18-20]. This type of intervention is usually designed to be transdiagnostic, easily adaptable in different settings, deliverable by nonspecialists, and based on sound evidence-based psychological principles embedded in a self-help approach (guided or unguided). This approach has already been validated in various target populations such as health care workers, migrants, and refugees to promote psychological well-being and clinical interventions, demonstrating general levels of acceptability and potential effectiveness [20]. A first attempt was made to make the protocol web-based within the RESPOND (Improving the Preparedness of Health Systems to Reduce Mental Health and Psychosocial Concerns Resulting From the COVID-19 Pandemic) project, but the involvement of a human person, in the role of helper, was still necessary [21].

Cognitive behavioral therapy techniques, particularly those referring to third-generation therapy, are shown to be more suitable than other psychotherapeutic approaches to be transferred into low-intensity interventions delivered by nontraditional methods, such as eHealth applications. SH+ is based on the principles of acceptance and commitment therapy (ACT), a third-wave form of cognitive behavioral therapy [22,23], and is divided into 5 chapters that address 5 strategies for managing stress and promoting psychological well-being. The five chapters, later translated into sessions, are as follows: (1) grounding (mindfulness), (2) unhooking (defusion), (3) acting on your values (values-based behavioral activation), (4) being kind (gratitude), and (5) making room (acceptance).

In this study, “acceptability” refers to participants’ perceived appropriateness, satisfaction, and willingness to engage with the digital intervention, as captured through self-report measures of user experience (UX) and user engagement (UE), as well as through semistructured interviews. “Feasibility” is defined as the extent to which the digital delivery of the SH+ intervention can be successfully implemented in the target population, considering aspects such as ease of access, completion rates, and usability. Both constructs are aligned with widely accepted definitions in the implementation science literature [24,25].

In conclusion, this study protocol aims to make the virtual coach, ALBA (A Well-Being Assistant), platform (described in the following paragraphs) available to women by evaluating the acceptability and feasibility of delivering SH+ through an assistant created to deliver psychoeducational sessions through dialogues, video, audio, and images (ie, the 5 chapters of SH+), as well as representing a virtual coach who guides the user in carrying out assigned exercises and tasks to be done independently between sessions. Moreover, we acknowledge that not all individuals who give birth identify as women, and that the more inclusive term would be “birthing person.*”* However, because most cases involve women, we have chosen to use the term *women* in this context. When specifically referring to birthing persons, appropriate linguistic and content-related considerations will be applied. Nevertheless, future replications of this intervention may be revised to ensure greater inclusivity.

### Goal of the Study and Research Questions

#### Overview

This study is positioned within the design and development cycle of the obesity-related behavioral intervention trials (ORBIT) model [26], specifically the refine phase. It involves delivering material based on SH+ in text, audio, and video formats by a virtual coach (ie, a digital assistant, identified as ALBA) implemented within the TreC Ricerca (TreC Research) app.

This intervention is embedded into the framework of behavior change interventions [27], which aim to enable women to acquire adaptive strategies to improve or maintain their psychological well-being. It should be noted that the term intervention in this context is used to identify a tool (in this case, the virtual coach) that will periodically be offered in a predefined (ie, rule-based), structured, and ordered manner, as informational material that has been structured and reviewed by psychological professionals.

This intervention has been developed and implemented for research purposes. The final goal is to validate the digital health intervention targeting women’s psychological well-being during pregnancy by helping them manage stressful situations more effectively through modules based on the principles of ACT.

#### Primary Objectives

The primary objectives of this proof-of-concept study described in this protocol paper are as follows: (1) to exploratively investigate through self-report standardized questionnaires UX and UE, that is, women’s experience and engagement when interacting with the TreC Ricerca app and the virtual coach, ALBA, (refer to the Step 3: Participation section for detailed description of questionnaires) and (2) to assess the women’s UE with ALBA and the app through semistructured interviews and investigate their feelings and overall experience during the intervention.

#### Secondary Objective

The secondary objective is to assess the level of pre-post psychological well-being through administered self-report questionnaires at the beginning and the end of the intervention.

This represents a preliminary exploration of the potential impact of the intervention by assessing changes in psychological well-being through post self-report questionnaires administered at the beginning and end of the intervention. Although this does not constitute an efficacy evaluation per se, it aligns with the *refine* phase of the ORBIT model, where early signals of potential effectiveness may be explored alongside feasibility and acceptability. This preliminary assessment aims to inform the design of future pilot or randomized controlled trials.

### ALBA Intervention

#### Overview

The virtual coach, ALBA, will deliver an intervention based on ACT techniques to promote QoL and perceived psychological well-being and manage stressful situations for pregnant women. The module is self-administered and available for both Android (Google LLC) and iOS devices (Apple Inc). All the contents of the dialogues were developed by a group of researchers affiliated with the Digital Health Research of the Bruno Kessler Foundation of Trento (Fondazione Bruno Kessler, Trento [FBK]) and psychologists from the Istituto Pavoniano Artigianelli with specific communication skills, based on the SH+ manual [28]. Subsequently, materials have been revised by a psychologist from the psychology operating unit of the Azienda Provinciale per i Servizi Sanitari di Trento (Provincial Health Services Authority of Trento; APSS). The dialogues, videos, and audio tracks were developed using an educational approach, rather than an emergency management approach. In addition, the structuring of the material and delivery methods was supervised by professionals from the WHO Center for Research in Mental Health at the University of Verona, who have already carried out implementation and validation studies of SH+ nationally and internationally.

To ensure the best possible adaptation of the intervention to our target sample, we previously conducted a co-design study based on guidelines of a previous step of the ORBIT model [26]. This process involved domain experts, such as psychologists, SH+ experts, communication and usability experts, and a small sample of new mothers and clinicians (midwives and gynecologists). For further details, please refer to our previous study [29].

#### Technological Tools

The technological component of the study is based on TreC. This platform allows citizens of the Autonomous Province of Trento to access, manage, and share information on their health and well-being [30]. TreC stands for *cartella clinica del cittadino* (citizen’s medical record) and is a reliable and well-tested platform designed to be a “system of systems” rather than a simple data hub. The central pillar of the TreC platform is the role of the citizen or as the manager of their health-related data, as in the case of a personal health record. TreC is designed with a flexible architecture, which enables the collection and management of heterogeneous data and allows the development and use of further subsystems to provide additional and specific functions.

This research project uses an additional module of the TreC platform, TreC Ricerca. The app can be downloaded via a specific link sent directly to participants. Authentication should be carried out via 2-factor authentication (one-time password code), which is secure and General Data Protection Regulation–compliant.

#### SH+ Sessions

**Figure 1:**
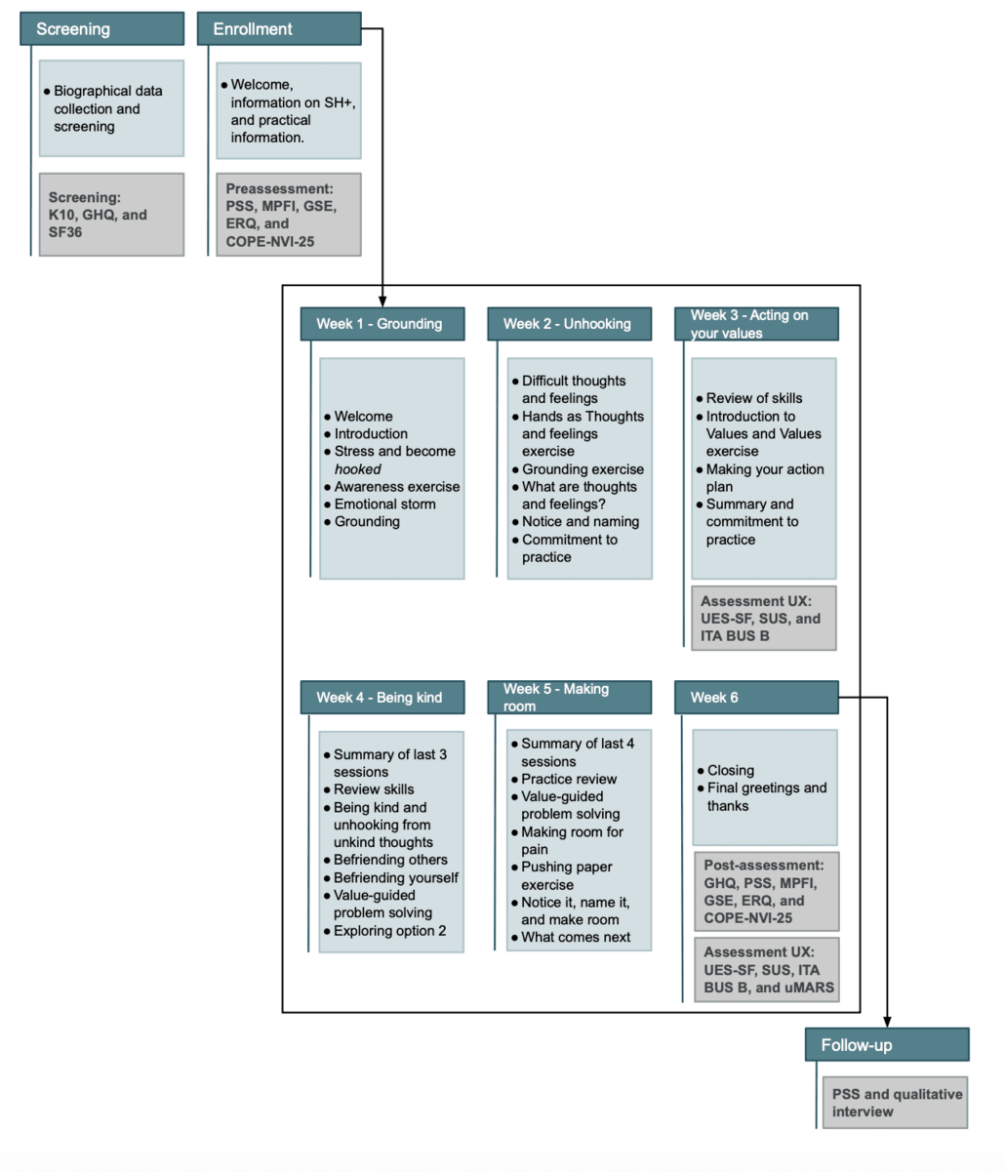
Graphical representation of the conversational protocol delivered to women and its chronological structure. COPE-NVI-25: Coping Orientation to the Problems Experienced–New Italian Version (25-item); ERQ: Emotion Regulation Questionnaire; GHQ: General Health Questionnaire; GSE: General Self-Efficacy Scale; ITA BUS B: Italian version of the Chatbot Usability Scale (version B); K10: Kessler Psychological Distress Scale; MPFI: Multidimensional Psychological Flexibility Inventory; PSS: Perceived Stress Scale; SF-36: 36-Item Short Form Health Survey; SH+: Self-Help Plus; SUS: System Usability Scale; UES-SF: User Engagement Scale-Short Form; uMARS: User Mobile Application Rating Scale; UX: user experience.

SH+ intervention lasts 6 weeks, with 1 session of approximately 40 per week, which can be divided into 2 subsessions of 20 minutes each. Figure 1 shows a graphical representation of the conversational protocol delivered to women and its chronological structure.

This intervention aims to enable women to acquire healthy coping strategies in stressful situations to promote psychological well-being during and after pregnancy.

**Figure 2:**
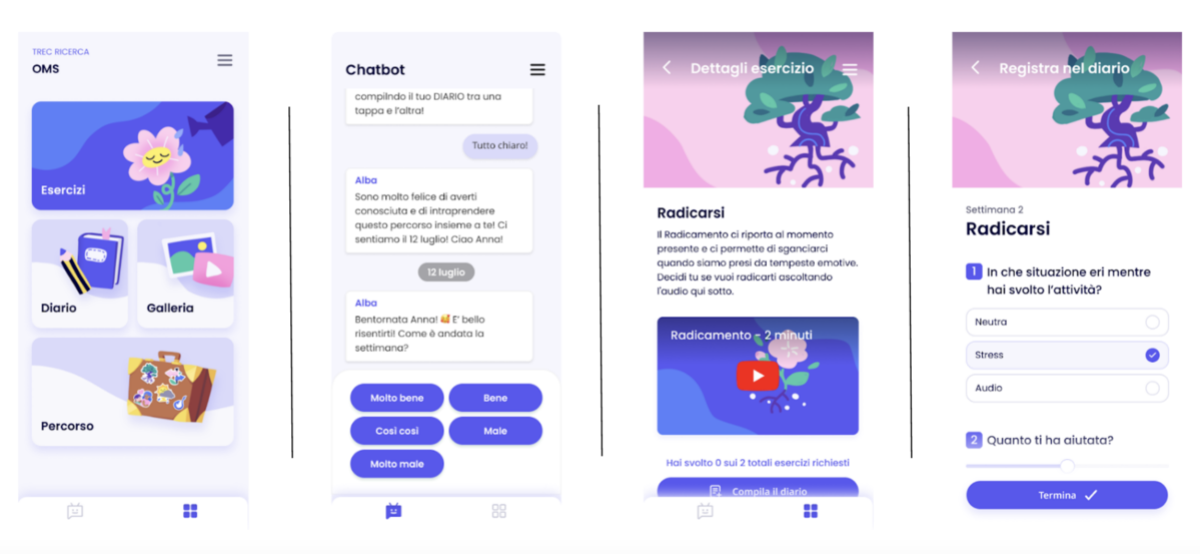
Mockups of the coach, ALBA (A Well-Being Assistant), within the TreC Ricerca app with some of its different sections.

The intervention was developed by referring to the SH+ program and adapting it to a psychoeducational course delivered via a virtual coach (refer to Figure 2 for some examples). The contents are presented through a gradual path that will enable the woman to become aware of herself and the present moment, accept and normalize internal states, cope with stress, and perceive an increase in her psychological well-being. In addition, the woman will be asked to perform tasks independently and fill out her diary daily.

## Methods

### Design and Study Plan

ALBA is designed to interact with pregnant women for 6 weeks, starting between weeks 14 and 26 of pregnancy. This period was chosen because the risk of miscarriage was much lower [31], a factor that could, of course, affect stress levels [32]. This will also ensure that we can complete the study before the due date of the participants. Weekly sessions are followed by the virtual coach’s instructions to independently perform some simple exercises and fill in the personal diary. The woman can choose the best day and time to interact with ALBA and perform exercises.

At the beginning of the pathway, ALBA will administer 6 self-report questionnaires, described in the Data Collection section, to establish a baseline for the examined psychological variables. After the data collection phase, the module will be administered, and its contents will be presented in different formats (ie, text, images, audio, and video).

An additional round of questionnaire administration is foreseen at the end of the intervention to assess potential changes in terms of psychological well-being and QoL perceived by the women. Four questionnaires will be administered to assess usability. To ensure the collection of all the necessary data, reminders were scheduled to complete the questionnaires 24, 48, and 72 hours after the first request. However, at present, there are no reminders for session completion.

Two months after giving birth, women from whom consent has been obtained will be invited to a semistructured interview to discuss their experience using ALBA and the app and to collect a more in-depth understanding of their experiences during the psychoeducational process. The 2-month postpartum period was chosen for both organizational convenience and methodological considerations. The expert group identified this timeframe to find a balance between scientific appropriateness and practical arrangements of the interviews, avoiding a prolonged span after the end of the intervention while ensuring that interviews with the participants were not conducted too close to the week of childbirth. The underlying assumption is that the period immediately following childbirth may be a logistically and emotionally complex time for the family, with a particular focus on the mother’s availability and experience.

### Participant Recruitment and Withdrawal

Recruitment will occur within the group of pregnant women between the 14th and 26th weeks of gestation attending the pregnancy care services of the APSS in Trentino.

Midwives from the APSS hospital and territorial service were involved in the study to identify women potentially eligible to participate.

The inclusion and exclusion criteria for women to participate in the study are provided in Textbox 1.

**Figure 3:**
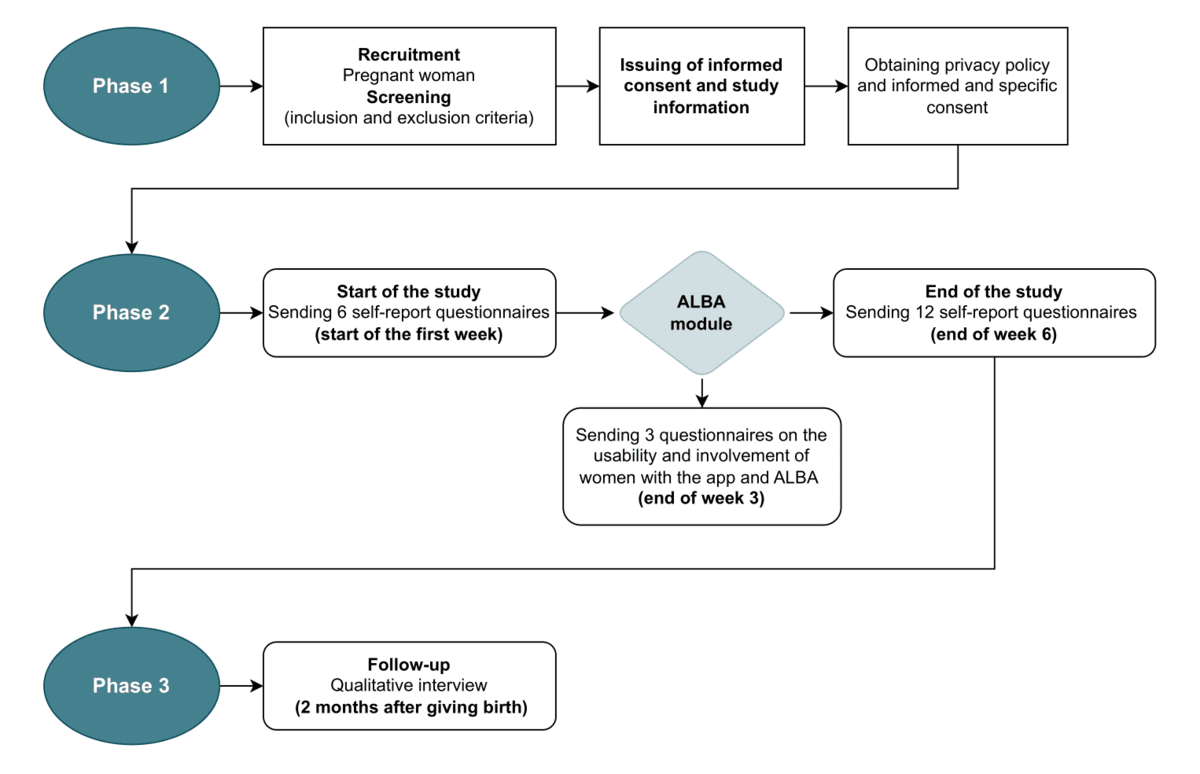
Flowchart of the research project procedure. ALBA: A Well-Being Assistant.

Figure 3 shows a flowchart of the research project procedure.

**Inclusion and exclusion criteria.**

**Inclusion criteria**

1. Be pregnant
2. Be in a gestational state between the 14th and the 26th week
3. Be aged ≥18 years
4. Have a smartphone with internet access, be able to download the app, and be able to use it
5. Be a resident of the Autonomous Province of Trento
6. Know and understand the Italian language
7. Have a stress level score, as measured by the Kessler Psychological Distress Scale, between 15.9 and 29.9
8. Have a general well-being score, as measured by the General Health Questionnaire, ≥3
9. Obtain a score on the Mental Health scale of the 36-Item Short Form Health Survey between 30 and 80.

**Exclusion criteria**

1. Patients unable to provide informed consent, which is a prerequisite for participation in the study
2. Inadequate understanding of the Italian language
3. Substance addiction or in recovery for 1 year
4. Suicidal tendencies
5. Depression or other psychiatric diagnoses
6. Women undergoing medically assisted procreation (due to the increased risk of anxiety and depressive states, ascertained in the literature)
7. Already being placed, followed, or taken care of on a psychological or psychotherapeutic path at the time of recruitment

### Sample Size

This research project is designed as a proof-of-concept study, where a limited sample is sufficient to achieve the intended aims. It was calculated that if nonparametric statistics are conducted (assuming the distribution is not normal), the sample should consist of 24 pregnant women with Bonferroni correction (α=.025). The sample size and power for nonparametric tests (Kruskal-Wallis and Wilcoxon post hoc tests) were calculated according to method reported by Noether [33]. If parametric statistics are conducted (assuming the normal distribution), the sample should consist of 41 pregnant women with Bonferroni correction (α=.025). The sample calculation was further confirmed with the

Power Analysis and Sample Size (NCSS LLC) and Stata (StataCorp LLC) programs, for which the resulting numerosity is 41 participants, assuming null hypothesis (Ho):d=d0, alternative hypothesis (Ha):d≠d0, and considering as study parameters α=.025; mean of group 1 (ma1): 0.000; power: 0.800; mean of group 2 (ma2): 1.000; delta (difference of means, delta): 0.500; expected difference (da): 1.000; and SD of difference (sd_d): 2.000.

Thus, by a power level set at 0.80, a significance level of .025, and considering a 20% potential dropout rate, an estimated 50 pregnant women are needed for recruitment to conduct the study.

### Ethical Considerations

The ethics committee of the APSS approved this study (17241). This study was approved by the ethics committee of the APSS (17241). Recruited women who meet the inclusion criteria and consent to participate will be enrolled in the research. Participants are enrolled voluntarily and do not receive any form of compensation. Their data are pseudonymized using unique codes, and all analyses will be conducted on aggregated data, ensuring that no individual can be identified or traced.

All women who decide to participate in the study will be asked to sign an informed consent form at the time of enrollment after a careful explanation of the project; its aims; how the data will be collected, managed, and processed; the level of involvement required; and the duration of the research, as well as any confidentiality issues. The woman will also be informed of the possibility of quitting the study at any time she wishes without any explanation and that this option would not impact the quality of care or interfere with her course of treatment.

The study will be explained partially by the midwives. In addition, a video recorded by the research team explaining the study, the purpose, and the woman’s involvement in simple, understandable words will also be available. The participants will be informed of the study’s results through the app.

Regarding data protection, a privacy policy will be provided according to articles 13 and 14 of the Regulation EU 2016/679 of the General Data Protection Regulation, explaining the purposes and legal grounds of the data processing, how the personal data will be collected, their categories, how it will be managed, including the data retention period, the obligations of the data controllers, and the rights of the participants who provide data. Personal data are processed for specific research purposes within the scope of public interest tasks of the data controllers. Informed, freely given, voluntary, and explicit consent will be collected regarding the processing of particular categories of personal data (ie, data concerning health and self-reported behavioral habits in the area of lifestyle health). Moreover, specific consent will be requested regarding the possibility of contacting the participant via telephone to conduct the research interviews.

All data collected will be kept confidential and managed by authorized persons. Data necessary for evaluating the study objectives will be pseudoanonymized before processing. Copies of the study information and the privacy policy will be issued to the participant and will always be available in a dedicated section of the app.

The study manager will produce a research report and report the data responsibly and consistently. Personal data will neither be disclosed nor disseminated except in anonymized or aggregated form for publishing purposes. The publication of data from this study will take place independent of the results obtained. The transmission or dissemination of the data, through scientific journals and presentations at congresses, conferences, and seminars, will take place exclusively in anonymous form using only aggregated data that prevents any identification of the participants, even indirectly. This sharing is expected within 1 year after the conclusion of the study.

### Study Outcomes

#### Primary Outcome

The primary outcomes are UX and UE. They will be assessed using 4 questionnaires, namely the User Engagement Scale-Short Form (UES-SF) [34], the System Usability Scale (SUS) [35], the Italian version of the Chatbot Usability Scale (version B; ITA BUS B) [36], and the User Mobile Application Rating Scale (uMARS) [37], which will be administered at different time slots throughout the study, in particular at the end of the week 3 and at the end of the study (ie, week 6).

The end points that will be measured are the scores of the individual items of the questionnaires (UES-SF, SUS, ITA BUS B, and uMARS), the average score of the questionnaires, and the difference between the average score values of the individual questionnaires at different survey times.

UX refers to the overall perceptions and responses that arise from interacting with a product or system, with reference to aspects such as usability, aesthetics, and functionality [38]. UE describes the user’s cognitive, emotional, and behavioral involvement during this interaction [39]. The chosen instruments capture complementary dimensions of UX and UE, including usability, aesthetics, engagement, and satisfaction (for detailed description, refer to Step 3: Participation section). These were selected due to their widespread validation and adaptation in human-computer interaction [40] and their focused use for mobile health app evaluation [41], particularly for chatbot evaluation [36]. Their dimensions are theoretically linked to constructs from cognitive psychology [42] and UX design [43], offering a deep understanding of how users interact emotionally and cognitively with digital products [35,44].

The evaluation of the experience of using the app and the virtual coach (ALBA) and the use of the intervention itself will also be assessed through the semistructured interviews conducted 2 months after childbirth. In this case, qualitative data will be collected to enrich the information related to the primary outcome.

The platform adopted for the intervention allows for recording activity logs, enabling proper mapping of patients’ actions and experiences. This covers a range of logs, assessing whether the patient is appropriately exposed to the intervention protocol while recording use patterns (eg, session execution time, number of accesses, and use patterns of different features). Among the variables considered are any missed sessions or interruptions. These data will be correlated with acceptability scores during the analysis phase. In case a patient does not complete activities and fails to fill in specific questionnaires (leading to data missing), particular pieces of information on patients’ experiences and platform use will be collected during the scheduled postintervention interview sessions.

#### Secondary Outcome

The variable expressing the “psychological well-being” outcome will be assessed by administering 6 self-report questionnaires at the beginning (ie, week 0) and the end (ie, week 6) of the module (refer to Table 1 for a detailed overview of the questionnaire adopted). The end points that will be measured are as follows: a score of the individual items of the questionnaires, the average score of the questionnaires, and the difference between the average score values of the individual questionnaires in the 2 survey times.

**Table 1:** Summary of the questionnaires administered and their timing.

Questionnaires	Screening	At the beginning of the study (week 0)	At the end of week 3	At the end of the study (week 6)	Follow-up (2 months after birth)
K10^a^	✓				
GHQ^b^	✓			✓	
SF36^c^	✓				
PSS^d^		✓		✓	✓
MPFI^e^		✓		✓	
GSE^f^		✓		✓	
ERQ^g^		✓		✓	
COPE-NVI-25^h^		✓		✓	
UES-SF^i^			✓	✓^j^	
SUS^k^			✓	✓^j^	
ITA BUS B^l^			✓	✓^j^	
uMARS^m^				✓^j^	
Qualitative interview					✓

^a^K10: Kessler Psychological Distress Scale.

^b^GHQ: General Health Questionnaire.

^c^SF36: 36-Item Short Form Health Survey.

^d^PSS: Perceived Stress Scale.

^e^MPFI: Multidimensional Psychological Flexibility Inventory.

^f^GSE: General Self-Efficacy Scale.

^g^ERQ: Emotion Regulation Questionnaire.

^h^COPE-NVI-25: Coping Orientation to the Problems Experienced–New Italian Version (25-item).

^i^UES-SF: User Engagement Scale-Short Form.

^j^These usability questionnaires will be administered the following day, so as not to burden the participant during completion.

^k^SUS: System Usability Scale.

^l^ITA BUS B: Italian version of the Chatbot Usability Scale (version B).

^m^uMARS: User Mobile Application Rating Scale.

These psychological instruments were selected for their widespread validation and adaptation in clinical and psychological research, as well as their relevance for assessing various aspects of mental health and coping in the context of digital health interventions. For example, the Kessler Psychological Distress Scale (K10) is a widely used screening tool for psychological distress, with strong validity across cultures, including Italian culture [45,46]. Furthermore, the Multidimensional Psychological Flexibility Inventory (MPFI) and the Perceived Stress Scale (PSS) are highly relevant for examining psychological flexibility and perceived stress, 2 critical constructs for understanding emotional and behavioral responses in mobile health contexts [47-49], (for detailed description of all questionnaires, refer to Step 3: Participation section).

In the framework of this study, these tools are adopted to assess early signals of potential effectiveness of the intervention while assessing UX and UE.

### Data Collection

#### Step 1: Data Collection

The first phase of participant recruitment will take place in collaboration with midwives. In this first phase, the clinical professionals will collect and send the sociodemographic data and email addresses of possible participants (ie, those women who meet inclusion criteria 1 to 6 and have no exclusion criteria) to the FBK researchers, after obtaining informed consent and explaining the study, its purposes, and the need for verification of the characteristics required by inclusion criteria 7 to 9.

Midwives will collect sociodemographic data using a prestructured form. Each woman will be assigned a unique alphanumeric code. The sociodemographic data collection form and the data collected during the study will be stored separately. In addition, data will be pseudonymized to ensure confidentiality.

The parameters requested from pregnant women will be (1) date of birth, (2) expected date of delivery, (3) educational level, (4) occupation, (5) marital status, (6) the number of pregnancies and deliveries, and (7) partner’s occupation, if any.

#### Step 2: Screening

To fulfill the verification of inclusion criteria 7 to 9, the researchers will contact the participants via email and administer the screening questionnaires in Table 1 via the LimeSurvey platform [50]. This platform complies with current data protection requirements and regulations. After the results of the screening questionnaires and the different sociodemographic inclusion criteria have been processed, the women will be contacted again. The following scenarios will open up. First, the participant does not meet the inclusion criteria by manifesting a markedly positive psychological well-being status (stress level, as measured by the K10, <15.9; a general well-being score, as measured by the General Health Questionnaire (GHQ), <3; and a well-being level >80 on the “mental health” scale of the 36-Item Short Form Health Survey (SF-36). In this case, the woman may not be enrolled in the study, and the reasons for exclusion and the results obtained on the screening questionnaires will be returned by the research psychologists only upon the participant’s request. Second, the participant does not meet the inclusion criteria by manifesting a markedly negative psychological well-being status (stress level measured by the K10>29.9 and obtaining a well-being level <30 on the “mental health” scale of the SF-36). Even in the latter case, the woman may not be enrolled in the study, and the reasons for exclusion from the study and the results obtained on the screening questionnaires will be returned by the research psychologists only upon the participant’s request. In addition, indications of territorial services to which to turn for problems that have arisen will be appropriately provided, again only at the participant’s request. This is so that a structured and agreed system of possible access to a territorial service of qualified psychological support can be suggested to the woman concerned, who will provide intake when appropriate. Third, the participant meets the inclusion criteria and is enrolled in the study.

#### Step 3: Participation

##### Overview

Women who meet the inclusion criteria and provide consent to participate in the study are enrolled in the study. All women who decide to participate in the study will be asked to sign an informed consent after a careful explanation of the study, as described in the Participant Recruitment and Withdrawal section.

Participants will also be asked if they would like to take part in a qualitative interview 2 months after delivery. In case of positive feedback, their telephone number will be collected to facilitate future contact and follow-up interviews. Women who provide consent to this interview will be sent (again via the app) a questionnaire 1 month after the expected date of delivery in which the following will be asked: (1) actual date of delivery, (2) type of delivery, (3) sex of baby, (4) name of baby, (5) mode of breastfeeding, and (6) date and time when the woman wishes to be contacted for the interview.

As shown in Table 1, the psychoeducational intervention involves completing self-report questionnaires delivered at the beginning, during, and at the end of the interaction with ALBA, in addition to the initial screening phase. Completing the instruments takes about 20 minutes. Specifically, 5 questionnaires will be administered at the beginning and 6 at the end of the intervention to investigate stress, self-efficacy, emotional regulation, psychological flexibility, coping strategies, and psychological well-being. None of these questionnaires has diagnostic purposes, so they will not be used to make diagnoses of psychopathology but only to collect descriptive data. Women with psychopathology will be excluded a priori from participation in the study (as defined in the Participant Recruitment and Withdrawal section). Four questionnaires will be delivered during the course to assess the usability and engagement of the woman with the app and ALBA. Specifically, usability questionnaires will be offered at the end of weeks 3 and 6 (ie, the end of the study).

A detailed description of each instrument that will be administered is presented in subsequent sections.

The Coping Orientation to Problems Experienced (COPE) [51] is a shortened version of the COPE Inventory, designed to assess various coping strategies individuals use to deal with stress. This 25-item questionnaire explores strategies, such as active coping, planning, and acceptance, providing a complex picture of an individual’s coping behavior. It uses a Likert scale from 1 (never do it) to 4 (do it often), with a minimum score of 25 points and a maximum of 100. Foà et al [52] translated and validated the questionnaire in Italian, showing good psychometric characteristics.

The Emotion Regulation Questionnaire [53] is a 10-item scale designed to measure the tendency to use 2 emotional regulation strategies as cognitive reappraisal and expressive suppression. Respondents answer each item on a 7-point Likert-type scale ranging from 1 (strongly disagree) to 7 (strongly agree). Balzarotti [54] translated and validated the questionnaire in Italian, showing good psychometric characteristics. In this study, Cronbach α was 0.81 for cognitive reappraisal and 0.84 for expressive suppression.

The GHQ is a self-assessment instrument designed to identify mental health disorders and monitor general psychological well-being. Developed by Goldberg and Blackwell [55], the GHQ is widely used in clinical, research, and public health settings. The 12 items of the questionnaire are formulated to investigate how the individual has been feeling recently, with reference to psychological symptoms (such as anxiety and depression), ability to cope with everyday situations, sleep disturbances, and somatic symptoms. The response is based on a 4-point Likert scale, ranging from “much better than usual” to “much worse than usual” with a maximum score of 36. Fontanesi et al [56] translated and validated the questionnaire in Italian, showing good psychometric characteristics.

The General Self-Efficacy Scale (GSE) [57] is a psychometric instrument designed to assess an individual’s general perception of self-efficacy, that is, confidence in one’s ability to organize and perform the actions necessary to handle potentially stressful or difficult situations. It consists of 10 statements on which respondents must express their level of agreement on a Likert scale ranging from 1 (not true for me) to 4 (exactly true for me). Items on the scale explore various aspects of self-efficacy, such as problem-solving ability, overcoming obstacles, and handling unexpected situations. The total score, obtained by summing the scores of all items, reflects the individual’s overall perceived level of self-efficacy and can range from 10 to 40. The GSE scale was translated into 28 languages. Scholz et al [58] examined the instrument’s psychometric properties in 25 countries, concluding that the construct of perceived self-efficacy is international and that the GSE is an equivalent measure across different cultures. Cronbach α ranged from 0.79 to 0.90. The Italian adaptation of the GSE scale was developed by Sibilia et al [59].

The K10 is a widely used screening instrument to measure psychological distress. Developed by Kessler et al [45], the K10 is a short questionnaire consisting of 10 items that investigate the frequency of psychological symptoms experienced by the individual in the past month, such as nervousness, sadness, fatigue, and feelings of hopelessness. Each question is scored on a 5-point scale, ranging from “not at all” to “all the time.” Total scores can range from a low of 10 to a high of 50, with higher scores indicating a higher level of psychological distress. The Transcultural Mental Health Center also makes available the validated version of this questionnaire in Italian [46].

The MPFI [47] is an assessment tool that measures psychological flexibility on several dimensions. It investigates how people adapt their thoughts and behaviors in response to changing situations and challenges. The MPFI focuses on various aspects of psychological flexibility, including openness to experiences, present-moment awareness, and value-driven action. The 12 dimensions of flexibility and inflexibility (according to the hexaflex model) are assessed through 60 Likert scale items from 1 to 6. The Italian adaptation of the MPFI scale was developed by Landi et al [48].

The PSS [49] is a self-report questionnaire used to measure perceived stress. Specifically, it aims to determine the degree to which situations are perceived as stressful in a person’s daily life. Ten 5-point Likert scale questions investigate thoughts or feelings one has had in the past month about certain events, such as how often one has felt unable to control important things in one’s life. The Italian version of the PSS-10, translated by Andrea Fossati and obtained from the Carnegie Mellon University website [60], was used.

The SF-36 [61] is the most widely used instrument for measuring general health status. It includes 8 scales: physical functioning (10 items), limitations due to physical health (4 items), pain (2 items), perception of general health (5 items), energy and fatigue (4 items), social activities (2 items), limitations due to emotional problems (3 items), and emotional well-being (4 items). The 36th item differentially assesses the change in health status (2 item) from the previous year. For each dimension, question scores are coded, summed, and transformed into a scale ranging from 0 (worst possible health status) to 100 (best possible health status). The SF-36 has been used in large population studies and in many different clinical conditions, showing excellent psychometric properties. It has been translated and validated in several languages, including Italian [62].

The ITA BUS B [36] is designed to assess users’ ease of use, effectiveness, and satisfaction in interacting with chatbots. This tool is structured around 11 items that cover various aspects of the UX with chatbots, aiming to provide an in-depth, multidimensional assessment of their usability. The items involve responses on a 5-point Likert scale, where 1 equals “strongly disagree” and 5 equals “strongly agree,” thus scoring from 11 to 55.

The SUS [63] is a quick and reliable tool for assessing system usability. It consists of 10 items with Likert-type responses from 1 to 5. The questionnaire, developed by Brooke [35], provides an overall score that reflects the ease of use and applicability of a system or product. This score can range from 10 to 50. A document with printable SUS questions is available online [63,64].

The UES-SF [34] is a short self-report questionnaire to assess UE with a digital solution. This measure includes 12 items based on a 5-point Likert scale, ranging from 1 (strongly disagree) to 5 (strongly agree). The questionnaire consists of 4 factors: (1) focused attention, which indicates the feeling of being immersed in the interaction; (2) perceived usability, which is the negative effect experienced due to the interaction and the effort expended; (3) aesthetic attractiveness, which represents the graphical and visual appeal to a digital solution; and (4) the reinforcement factor (reward). The latter is a single factor that includes duration, which evaluates the overall success of the interaction; novelty, which examines the general interest related to the interaction with a digital solution; and, finally, the perceived engagement factor, which evaluates the overall enjoyment of the interaction. This questionnaire was not available in Italian and was, therefore, translated through the back translation procedure.

The uMARS [65] is a tool for assessing the quality and functionality of mobile apps. It is characterized by a 20-item measure that includes 4 objective quality subscales (engagement, functionality, aesthetics, and information quality) and 1 subjective quality subscale rated on a 5-point Likert scale, ranging from 1 (poor) to 5 (excellent). The total and subscales scores have very high coefficients (0.90 and 0.78-0.80, respectively). The scale has also been validated in the Italian context [66].

##### Qualitative Interview

A chartered psychologist (SR) from FBK will conduct the interviews. The interviews are structured with ad hoc items to purposively capture specific characteristics of the study and participants’ experiences [67]. They will be carried out 2 months after the delivery, last approximately 20 minutes, are subject to the woman’s consent, and will be audio-recorded to allow subsequent analysis.

The platform adopted for the intervention allows for recording activity logs, enabling proper mapping of patients’ actions and experiences. This covers a range of logs, assessing whether the patient is appropriately exposed to the intervention protocol while recording use patterns (eg, session execution time, number of accesses, and use patterns of different features). Among the variables considered are any missed sessions or interruptions. These data will be correlated with acceptability scores during the analysis phase. In case a patient does not complete activities and fails to fill in specific questionnaires (leading to data missing), particular information on patients’ experiences and platform use will be collected during the scheduled postintervention interview sessions.

#### Data Analysis

##### Quantitative Analysis

Statistical data processing will be conducted using the software R (version 4.0.0; R Foundation for Statistical Computing), SPSS Statistics (IBM Corp), and Stata (version 17; StataCorp LLC).

Categorical variables will be summarized through absolute and percentage frequency distributions, and quantitative variables through appropriate centrality and variability indices.

Descriptive analyses will be calculated for both psychological variables (stress level, anxiety level, depression level, emotional regulation, psychological flexibility, coping strategies, well-being, and QoL) and variables on usability (UX) and UE. These analyses will be performed on the variables analyzed at the beginning, during, and at the end of the interaction with ALBA.

The relationships between variables will be analyzed mainly through ad hoc statistical tests, such as the chi-square test, Fisher exact test, 2-tailed *t* test for paired data, Wilcoxon nonparametric tests, and the sign test (based on assessment of compliance with assumptions), to understand the differences between the beginning and the end of the course in the same study sample with respect to the variables under investigation.

In addition, univariate logistic or multinomial regression models will be presented. Finally, to eliminate possible confounders, multiple regression models will be proposed in which the effects of the explanatory variables on the outcome variables will be adjusted for possible confounders. For each analysis, statistical significance will be found with a *P* value of ≤.05. It is also planned to use McNemar test [68] and Cochran Q [69] to evaluate paired qualitative data.

##### Qualitative Analysis

For the analysis of the semistructured interviews, a text mining approach [67] will be used to extract the responses that appear repeatedly in the interviews. To support the analysis, specific software tools, such as NVivo (version 14; Lumivero), could be adopted [70]. The analysis will involve identifying frequently occurring words or phrases to explore dominant lexical patterns. Sentiment and polarity will also be assessed by classifying the emotional tone of texts as positive, negative, or neutral. In addition, meaningful excerpts (quotes) will be extracted, and all results will be interpreted in the context of the relevant literature. The interviews will be conducted regarding the women’s experience of using ALBA and how they felt throughout the process (Multimedia Appendix 1).

## Results

Recent literature underscores a growing interest in interventions based on ACT, primarily due to their demonstrated effectiveness in alleviating anxiety, depression, and fear of cancer recurrence, as well as in enhancing psychological flexibility and overall QoL. Core ACT techniques, such as mindfulness and cognitive defusion, support the management of anxiety and depressive symptoms by promoting emotional regulation and stress management through acceptance-based approaches. In addition, by encouraging value-driven actions, ACT is expected to foster improvements in well-being and life satisfaction. In line with these goals, this study includes key psychological variables—psychological flexibility (MPFI), self-efficacy (GSE), coping strategies (COPE) New Italian Version, 25-item), and emotion regulation (Emotion Regulation Questionnaire) as predictors, to provide a comprehensive assessment of ACT’s impact during different phases of oncological treatment.

From a technical perspective, collaboration with computer science experts will continue throughout the study to ensure the app’s structure and design are optimized. We have drawn on previous internal usability evaluations and a co-design study [24] to guide the app’s development and refine its features ahead of this pilot phase. During implementation, any new technical issues, such as bugs or glitches, will be monitored and addressed in real time. The primary objective of this feasibility study is to identify and resolve any usability challenges before advancing to a larger-scale trial. Its findings will guide future decisions about scaling the intervention or launching a more extensive efficacy study.

Key benchmarks for evaluating feasibility will include UE, retention and dropout rates, significant improvements in psychological measures (as mentioned earlier), and feedback on the app’s acceptability and usability. These data will be gathered through self-report questionnaires and technical assessments such as bug tracking. Should these benchmarks be achieved, the study will move toward a broader trial. Moreover, results from this phase will inform potential refinements to both the intervention and study design, ensuring they align more closely with the needs of the target population in terms of content, UX, and delivery methods.

Preliminary data analysis is scheduled for early October 2025, while final results will be available at the end of the study (first half of 2026). The results will be published within 1 year after the study’s conclusion.

## Discussion

### Anticipated Findings

This study aims to investigate and evaluate women’s experience and engagement with the TreC Ricerca app and the virtual coach dedicated to the stress intervention (ALBA) and assess the level of pre-post psychological well-being.

First, we expect to collect relevant feedback and suggestions from the women to improve the structure of the intervention, making it more engaging and improving the interaction. Second, we intend to find differences in terms of improved pre-post psychological well-being. However, we are aware that this change may not be related to the intervention itself and that the evaluation of effectiveness will be carried out through a randomized clinical trial at a later stage.

All results will be reported and adequately discussed through a comparison with the relevant literature.

### Limitations

This study has several limitations that should be considered and resolved during the implementation of future studies. As mentioned earlier, it is crucial to consider that this study does not aim to evaluate the effectiveness of the intervention, which is why a control group is not planned. It will be crucial to involve a control group to assess the actual effectiveness in a randomized controlled trial. In addition, this study involves several pregnant women from the Autonomous Province of Trento, Italy. It would be interesting to extend the study to women from other regions in Italy as well.

It is important to emphasize that ALBA might be a valuable technological support in providing regular psychoeducational services for women during pregnancy. Moreover, the literature has shown that women during the perinatal period indicated a preference for online support, suggesting that the implementation of digital interventions can overcome barriers to social stigma and seeking help [71,72]. Finally, it is crucial to remember that this technological support (ALBA and TreC Ricerca app) is not a substitute for clinical and medical pathways and in-life medical support; instead, it is an additional element in promoting psychological well-being and healthy lifestyles during pregnancy.

If the results of this study are positive, we expect that, after evaluating its effectiveness, the intervention could be made available as a tool to support the psychological well-being of pregnant women.

### Conclusions

Existing literature indicates a preference for online support among women in the perinatal period, highlighting the potential of digital interventions to address barriers related to social stigma and seeking assistance. In this context, ALBA emerges as a possible valuable resource, providing consistent psychoeducational support for women throughout the course of pregnancy.

## Appendix

Multimedia Appendix 1 Questions posed to participants attending the evaluation.

## Abbreviations

ACT: acceptance and commitment therapy
ALBA: A Well-Being Assistant
APSS: Azienda Provinciale per i Servizi Sanitari di Trento (Provincial Health Services Authority of Trento)
COPE: Coping Orientation to Problems Experienced
FBK: Fondazione Bruno Kessler (Bruno Kessler Foundation)
GHQ: General Health Questionnaire
GSE: General Self-Efficacy Scale
ITA BUS B: Italian version of the Chatbot Usability Scale (version B)
K10: Kessler Psychological Distress Scale
MPFI: Multidimensional Psychological Flexibility Inventory
ORBIT: obesity-related behavioral intervention trial
PSS: Perceived Stress Scale
QoL: quality of life
RESPOND: Improving the Preparedness of Health Systems to Reduce Mental Health and Psychosocial Concerns Resulting From the COVID-19 Pandemic
SF-36: 36-Item Short Form Health Survey
SH+: Self-Help Plus
SUS: System Usability Scale
TreC Ricerca: Trec Research
UE: user engagement
UES-SF: User Engagement Scale-Short Form
uMARS: User Mobile Application Rating Scale
UX: user experience
WHO: World Health Organization

## Data Availability

The datasets generated and analyzed during this study are available from the corresponding author upon reasonable request.
