# Supplementary material for: A Chatbot-Based Version of a World Health Organization–Validated Intervention (Self-Help Plus) for Stress Management in Pregnant Women: Protocol for a Usability Study
Source: JMIR Res Protoc. 2025 Aug 1;14:e53891. doi: 10.2196/53891 (PMC12357124; doi:10.2196/53891)
Supplement: Multimedia Appendix 1 [file resprot_v14i1e53891_app1.docx]

**Table 1.** Questions posed to participants attending the evaluation

| **Topic investigated** | **Questions** |
| --- | --- |
| Set 1: Interaction | 1.1 How did you feel about the interaction with ALBA? Were there enough alternatives among the replies to ALBA?  1.2 What was the feature of the interaction that you liked the most? And the one you liked the least? |
| Set 2: Content delivery | 2.1 If something was not clear to you at first, do you find there is then a way to investigate the topic further?  2.2 Is the mode of communication (length of sentences, terms used) appropriate for the content?  2.3 Did you enjoy the multimedia content?  2.4 What do you think of the exercises?  2.5 What do you think about gamification aspects (e.g. stickers)? |
| Set 3: Involvement and constancy | 3.1 Was the communication with the chatbot engaging? Did it entice you to get involved and be consistent in activities?  3.2 Overall, was the intervention personalized for you? Give a level of personalization from 0 to 10.  3.3 Do you think that the reminders have helped you to be more consistent? |
| Set 4: Other | 4.1 Do you have any concerns?  4.2 Do you have any criticism?  4.3 Would you recommend this path to another woman in the same condition as you? Why?  4.4 Are you satisfied with the course you took? Was it useful to you?  4.5 Are you satisfied with the use of the app and its structure? |
